# Plasma Extracellular Vesicles-Derived miR-99a-5p: A Potential Biomarker to Predict Early Head and Neck Squamous Cell Carcinoma

**DOI:** 10.3389/pore.2022.1610699

**Published:** 2022-10-18

**Authors:** Qiang Huang, Yu-Jie Shen, Chi-Yao Hsueh, Yi-Fan Zhang, Xiao-Hui Yuan, Yu-Juan Zhou, Jiao-Yu Li, Lan Lin, Chun-Ping Wu, Chun-Yan Hu

**Affiliations:** ^1^ Department of Otorhinolaryngology, Eye & ENT Hospital, Fudan University, Shanghai, China; ^2^ Shanghai Key Clinical Disciplines of Otorhinolaryngology, Shanghai, China; ^3^ Department of Pediatric, Xinhua Hospital, Shanghai Jiaotong University School of Medicine, Shanghai, China; ^4^ Department of Pathology, Eye & ENT Hospital, Fudan University, Shanghai, China

**Keywords:** biomarker, HNSCC, HPV, plasma extracellular vesicles, miR-99a-5p

## Abstract

**Purpose:** This study aimed to investigate the applicability of plasma extracellular vesicles (EVs) miR-99a-5p as a potential head and neck squamous cell carcinoma (HNSCC) diagnostic biomarker.

**Methods:** The miRNA expression of HNSCC tissue and plasma EVs were profiled by small RNA sequencing. qRT-PCR was performed to detect miR-99a-5p expression in HNSCC (*n* = 93) and benign disease (*n* = 39) plasma EVs and formalin-fixed and paraffin-embedded (FFPE) tissue (*n* = 110). We constructed receiver-operating characteristic curves to investigate the diagnostic efficiency of plasma EVs miR-99a-5p.

**Results:** Tumor tissue exhibited lower miR-99a-5p than para-tumor tissue. Patients with high miR-99a-5p expression exhibited significantly more p16 positive status. In contrast, HNSCC plasma EVs harbored more miR-99a-5p than the benign disease group. Plasma EVs miR-99a-5p distinguished HNSCC with area under the curve (AUC) of 0.7494 (95% CI: 0.6692–0.8296; *p* < 0.0001), with 61.54% sensitivity and 75.27% specificity, respectively. Furthermore, plasma EVs miR-99a-5p also distinguished early HNSCC with AUC of 0.7394 (95% CI: 0.6284–0.8504; *p* = 0.0002), with 79.07% sensitivity and 61.54% specificity, respectively.

**Conclusion:** Plasma EVs miR-99a-5p is a potential biomarker for predicting early HNSCC.

## Introduction

Head and neck squamous cell carcinoma (HNSCC), a heterogeneous collection of malignancies, accounts for approximately 90% of all head and neck cancers and is closely associated with human papilloma virus (HPV) infection [1]. The epidemiology, pathophysiology, and response to treatment of HPV+ HNSCC differ sharply from that of HPV− disease. Overall, HPV positive is associated with more favorable clinical outcomes [2]. Notably, HNSCC patients are at a high risk of cervical lymph node metastases. Cervical lymph node involvement is a well-known prognostic marker for HNSCC [3], and the presence of positive lymph nodes is thought to be a predictor of poor patient outcomes [4]. This highlights the importance of the early diagnosis of HNSCC. However, no screening strategy has yet proven to be effective for the detection of early HNSCC.

Plasma extracellular vesicles (EVs) are increasingly being promoted as potential disease detection biomarkers [5]. The cargo carried by EVs mimics the content of the parental cells and could be secreted into the peripheral circulation where they could be detected [6]. Shortly after Valida et al. [7] reported that EVs, especially exosomes, contained RNA components in 2007, Taylor et al. reported the possibility of using the miRNA components of EVs to identify diagnostic markers for ovarian cancer [8]. Guo et al. discovered and validated plasma EVs miR-95-3p/miR-26b-5p and its combination with CA19-9 serum levels for distinguishing pancreatic ductal adenocarcinoma from chronic pancreatitis [9]. These findings suggest the potential of plasma EVs miRNAs as differential diagnostic biomarkers in tumor patients [10].

MiR-99a-5p acts as a tumor suppressor by inhibiting proliferation, migration, and invasion and has been found to be dysregulated in several tumors, including bladder cancer [11], esophageal cancer [12]and HNSCC [13]. Furthermore, miR-99a-5p can be detected in plasma EVs during the identification and evaluation of biomarkers in cancer patients [14, 15]. However, little is known about the diagnostic potential of plasma EVs miR-99a-5p in HNSCC patients.

Hence, we sought to investigate the applicability of plasma EVs miR-99a-5p expression levels as a minimally invasive HNSCC diagnostic biomarker.

## Methods and Materials

### Patient Tissue and Ethics Approval

HNSCC tumor and para-tumor fresh tissue (*n* = 3 pairs) and formalin-fixed and paraffin-embedded (FFPE) HNSCC tissue (*n* = 110) were obtained from patients diagnosed with HNSCC (mainly hypopharyngeal and laryngeal squamous cell carcinoma) pathologically after surgery from October 2019 to October 2021. Plasma samples were obtained from HNSCC patients (*n* = 93) and benign disease patients (*n* = 39) from October 2019 to October 2021. All patient samples were obtained from the Department of Otorhinolaryngology, Eye & ENT Hospital of Fudan University. All participants provided written informed consent forms. This study was approved by the Ethics Committee of the Eye & ENT Hospital of Fudan University (NO. 2018036).

### RNA Isolation and qRT-PCR

Total RNA was isolated from FFPE tissue with an RNeasy FFPE Kit (QIAGEN, Germany) and from fresh tumor tissue and plasma EVs with TRIzol reagent (Invitrogen, Thermo Fisher Scientific) according to the manufacturer’s instructions, measured by a microspectrophotometer Nanodrop 2000 (Thermo Fisher Scientific), with the ratio of OD260/OD280 > 1.8, and then reversed-transcribed using an Evo M-MLV Mix Kit with gDNA Clean for qPCR (AG11728, Accurate Biology, Hunan, China). The housekeeping genes U6 was used as internal references to normalize gene expression for miRNA. The primers were designed and synthesized by Sangon Biotech (Shanghai). The sequences of primers used are as follows: miR-99a-5p: 5′ AAC​CCG​TAG​ATC​CGA​TCT​TGT​G 3′; U6: 5′ GTG​CTC​GCT​TCG​GCA​GCA​CAT 3′. qRT-PCR was performed using SYBR® Green Premix Pro Taq HS qPCR Kit (AG11718, Accurate Biology, Hunan, China) with the ABI 7500 Real-Time PCR System (Life Technologies, Shanghai, China) using the 2^−ΔΔCt^ method.

### Isolation and Purification of EVs

Peripheral blood samples were obtained in an ethylene diamine tetraacetic acid (EDTA)-coated tube (BD Pharmingen, New Jersey, USA) before surgery. Hemocytes were separated by centrifugation at 2,000 g for 15 min at 4°C. The clear top layer was obtained by another centrifugation at 10,000 g for 30 min at 4°C. The EVs derived from the plasma samples were isolated with Total Exosome Precipitation Reagent (Invitrogen, Thermo Fisher Scientific) according to the manufacturer’s instructions.

### Confirmation and Characterization of Plasma EVs

The morphological characteristics, size distributions, and marker detection of EVs pellets were examined by transmission electron microscopy (TEM), nanoparticle tracking analysis (NTA), and immunoblotting analysis, according to the methods described in our previous studies [16, 17].

### Small RNA Sequencing and Data Analysis

Small RNA library preparation and sample sequencing were performed with the assistance of Beijing Novogene Co., Ltd., using an Illumina HiSeq^TM^ 2500 device. Total RNA from fresh HNSCC and para-tumor tissue (*n* = 3 pairs) and plasma EVs (*n* = 6 for HNSCC patients, *n* = 3 for benign disease patients) were concatenated with 5′ and 3′ adaptors. The quantity and integrity of RNA yield was assessed by using the Qubit®2.0 (Invitvogen, USA) and Agilent 2200 TapeStation (Agilent Technologies, USA) separately. After cDNA synthesis and PCR amplification, the cDNA library (18–40 nt) was obtained using an acrylamide gel purification method, and single-end sequencing was then performed. The raw data (Raw reads) obtained by sequencing were filtered first: the joints at both ends of the reads were removed, and the reads with fragment length <17 nt and low-quality reads were removed to complete the preliminary filtering of data and obtain high-quality data (Clean reads). The distribution map of genome-wide reads was obtained by comparing Clean reads with the reference genome, and the Clean reads were annotated by ncRNA classification. The miRNA expression were calculated by RPM (Reads Per Million) values (RPM=(number of reads mapping to miRNA/number of reads in Clean data)×10^6^). Differential expression between two sets of samples was calculated by edgeR algorithm according to the criteria of |log_2_(Fold Change)|≥1 and *p*-value < 0.05.

### Bioinformatics Analysis

The expression of miR-99a-5p was downloaded from the latest TCGA project (Data Release 32.0) *via* the Genomic Data Commons Data Portal [18] (https://portal.gdc.cancer.gov/). The expression values of genes from miRNA-seq data were scaled with log_2_(RPM +0.01). A Kaplan–Meier plot was used to assess the correlation between the expression of miR-99a-5p in HNSCC.

### Statistical Analysis

Statistical analysis was performed using the Mann–Whitney test to evaluate the differences between the two groups. The Chi-square testing was used for the statistical calculation of categorical data. Kaplan-Meier method was used to calculate the survival rate, and Log-rank (Mantel-Cox) test and Gehan-Breslow-Wilcoxon test were used to test the difference in survival rate. GraphPad Prism 8.0 was used for statistical analyses and scientific graphing. The results are expressed as mean ± standard deviation (SD). Receiver-operating characteristic (ROC) curves were performed by plotting the true positive (sensitivity) against the false-positive (1-specificity) rate, and the area under the curve (AUC) was calculated. Optimal cut-off values were established based on the highest value obtained in the ROC curve analysis according to the likelihood ratio. Differences were considered significant if the *p* value was <0.05 (*), <0.01(**), <0.001(***), or <0.0001 (****), as indicated in each figure legend.

## Results

### miR-99a-5p Is Downregulated in HNSCC Tissue and Is Associated With HPV Infection Status

**FIGURE 1:**
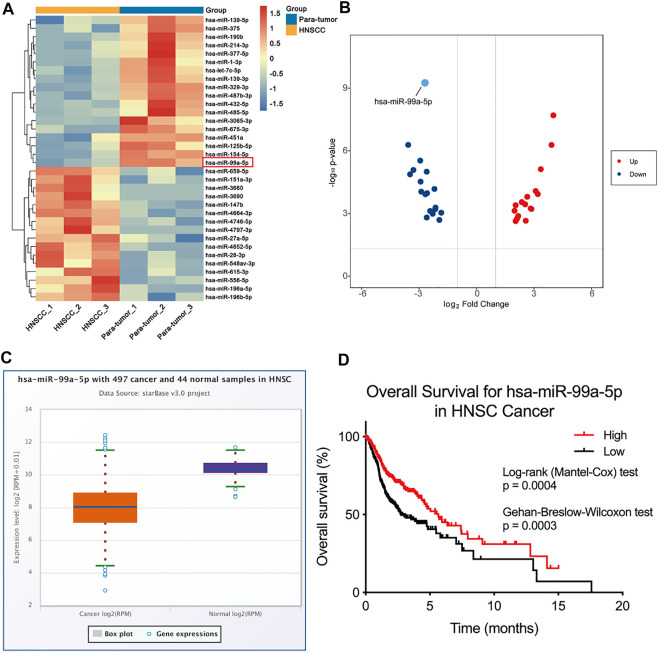
miR-99a-5p is downregulated in HNSCC tissue and is associated with HPV infection status. **(A)** Heatmap demonstrating the differentially expressed miRNAs (FDR <1%); those with difference of expression (more than 2-fold change) are shown (*n* = 3 pairs of tumor and corresponding para-tumor tissue). On the heatmap vertical axis are reported the names of the most significant miRNAs differentially expressed between the two groups, and on the heatmap horizontal axis are the sample identification numbers. **(B)** Volcano plots of miRNA-seq data demonstrating upregulated and downregulated genes in HNSCC tumor tissue compared to para-tumor tissue; red, upregulated; blue, downregulated. **(C)** The expression of miR-99a-5p in normal tissue (*n* = 44) and HNSCC tissue (*n* = 497) as downloaded from TCGA project *via* Genomic Data Commons Data Portal. The expression values of genes from miRNA-seq data were scaled with log2(RPM +0.01). **(D)** Overall survival of HNSCC patients in groups of high/low expression of miR-99a-5p (Log-rank test *p* = 0.0004, Gehan-Breslow-Wilcoxon test *p* = 0.003).

To depict the comprehensive miRNA profile with abnormal expression, miRNA sequencing was performed in three paired HNSCC tumors and para-tumor tissue. Differentially expressed miRNAs are listed in Figure 1A. miRNA-99a-5p was consequently selected for further study due to its high expression in para-tumors but low expression in HNSCC tumors (Figure 1B). Moreover, we verified the expression of miR-99a-5p in the Cancer Genome Atlas (TCGA) cohort. Data from 44 normal tissues and 497 HNSCC tumors confirmed that miR-99a-5p expression was higher in normal tissues than in tumor tissues, which was consistent with the sequencing results (Figure 1C). Furthermore, the Kaplan–Meier plot showed that low expression of miR-99a-5p in HNSCC tissues negatively affected the prognosis of HNSCC patients (*p* < 0.05) (Figure 1D).

We further determined miR-99a-5p expression in 110 HNSCC FFPE tissue using qRT-PCR. Detailed clinical and pathological data are depicted in Table 1. Overall, patients with high miR-99a-5p expression exhibited significantly more p16 positive status in pathological samples than did patients with low miR-99a-5p expression (*p* < 0.0001). No further significant associations were found between miR-99a-5p levels in HNSCC tissue and clinicopathological features (T stage, lymph node metastasis, clinical stage, tumor diameter, or pathological differentiation). We also found no difference in tissue miR-99a-5p levels in terms of prognosis (*p* = 0.3880) or recurrence (*p* = 0.3881) in HNSCC patients.

**TABLE 1:** Correlation between miR-99a-5p and clinicopathologic characteristics in 110 HNSCC FFPE tissue.

Parameter	All patients		High expression		Low expression		*p* value
	*n* = 110	%	*n* = 22	%	*n* = 88	%	
Age							0.1510
≤60	60	54.5	15	68.2	45	51.1	
>60	50	45.5	7	31.8	43	48.9	
Gender							>0.9999
Female	1	0.9	0	0	1	1.1	
Male	109	99.1	22	100	87	98.9	
Smoking history							0.3940
No	25	22.7	3	13.6	22	25	
Yes	85	77.3	19	86.4	66	75	
Drinking history							0.1247
No	35	31.8	10	45.5	25	28.4	
Yes	75	68.2	12	54.5	63	71.6	
Hypertension							0.6971
No	66	60	14	63.6	52	59.1	
Yes	44	40	8	36.4	36	40.9	
Diabetes							0.2253
No	99	90	18	81.8	81	92	
Yes	11	10	4	18.2	7	8	
p16							***<0.0001*** *
Negative	92	83.6	7	31.8	85	96.6	
Positive	18	16.4	15	68.2	3	3.4	
T stage							0.8485
T1+T2	52	47.3	10	45.5	42	47.7	
T3+T4	58	52.7	12	54.5	46	52.3	
Lymph node metastasis							>0.9999
Negative	20	18.2	4	18.2	16	18.2	
Positive	90	81.8	18	81.8	72	81.8	
Clinical stage							>0.9999
I + II	7	6.4	1	4.5	6	6.8	
III + IV	103	93.6	21	95.5	82	93.2	
Maximum tumor diameter (MTD)							0.5010
<3 cm	62	56.4	11	50	51	58	
≥3 cm	48	43.6	11	50	37	42	
Differentiation							0.3670
High+ High-moderate	92	83.6	17	77.3	75	85.2	
Moderate+ moderate-low	18	16.4	5	22.7	13	14.8	
Recurrence							0.3880
No	61	55.5	14	63.6	47	53.4	
Yes	49	44.5	8	36.4	41	46.6	
Death							0.3811
No	66	60	15	68.2	51	58	
Yes	44	40	7	31.8	37	42	

*p value was tested from Fisher’s exact test.

Collectively, these findings suggest that miR-99a-5p may play an important role in inhibiting the progression of HNSCC and is associated with HPV infection status.

### miR-99a-5p is Upregulated in HNSCC Plasma EVs

Considering the promising results obtained in tissue samples and the significant anti-tumor properties of miR-99a-5p previously described in HNSCC [19], we hypothesized that the tumor excreted cancer-suppressing miR-99a-5p into the peripheral circulation in the form of EVs to maintain survival [20]. To confirm this hypothesis and further describe the miRNA expression profile in plasma EVs of HNSCC patients, we isolated EVs from the plasma of six HNSCC patients and three benign disease patients, and then performed miRNA sequencing.

**FIGURE 2:**
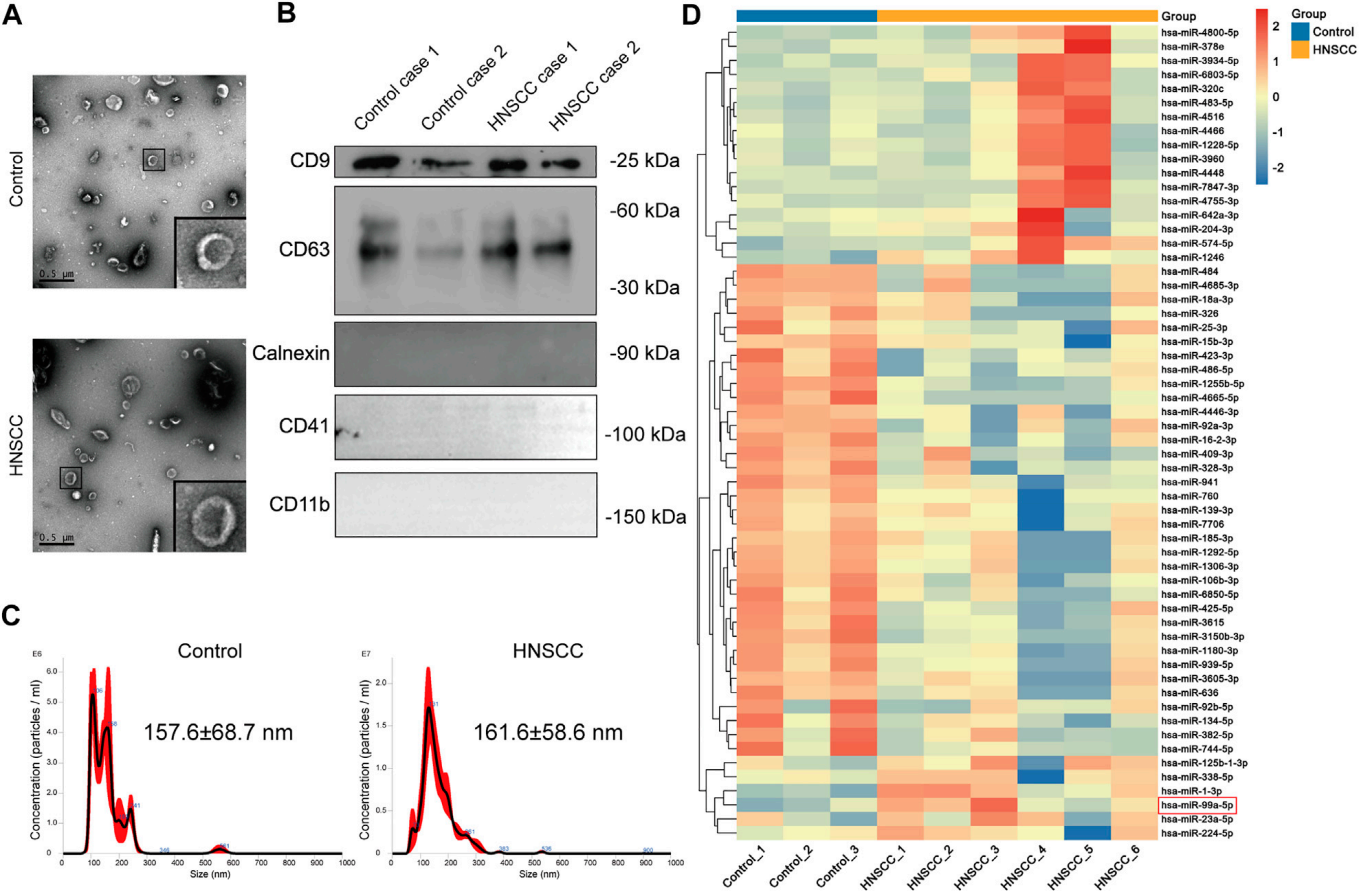
miR-99a-5p is upregulated in HNSCC plasma EVs. **(A)** The morphological characteristics of EVs derived from the plasma of HNSCC patients and benign disease patients (Scale bar: 0.5 μm). Partially amplified images are shown in the frame. **(B)** Immunoblotting analysis of the EVs markers of CD63 and CD9, and the negative marker Calnexin in plasma EVs derived from two cases of HNSCC patients and two cases of benign disease patients. **(C)** Size distribution and concentration of plasma EVs detected by NTA. **(D)** Heatmap demonstrating the differentially expressed miRNAs in plasma EVs (FDR <1%); those with difference of expression (more than 2-fold change) are shown (*n* = 3 benign disease patients; *n* = 6 HNSCC patients). On the heatmap vertical axis are reported the names of the most significant miRNAs differentially expressed between the two groups, and on the heatmap horizontal axis are the sample identification numbers.

We found that the EVs we isolated had a representative saucer-shaped vesicle structure with a double-layer membrane, according to the TEM analysis (Figure 2A). We further verified the expression of EVs surface markers CD63 and CD9, together with the negative marker Calnexin. Furthermore, plasma EVs did not express platelet and immune cell marker genes CD41 and CD11b (Figure 2B). The NTA results showed that the average size of the EVs was 157.6 ± 68.7 nm for EVs derived from patients with benign disease and 161.6 ± 58.6 nm for HNSCC patients-derived EVs (Figure 2C). These results confirmed that the EVs we isolated had representative morphology, appropriate size distribution, and precise surface markers typical of EVs.

Further, we identified several miRNAs, including miR-1228-5p and miR-4466, that were highly expressed in the plasma EVs of HNSCC patients through miRNA sequencing. Clinicopathological features of HNSCC patients undergoing plasma EVs miRNA sequencing are summarized in Table 2. Surprisingly, we found that, contrary to the miR-99a-5p level in tissue, miR-99a-5p expression levels in circulating plasma EVs were significantly higher in HNSCC patients than in benign disease controls (Figure 2D).

**TABLE 2:** Clinicopathological features of HNSCC patients undergoing plasma EVs miRNA sequencing.

No.	Gender	Age	Tumor type	T stage	N stage	Clinical stage	Pathological differentiation	p16 status
1	Male	73	LSCC	3	1	III	Well-moderately	Positive
2	Male	74	LSCC	3	1	III	Well-moderately	Positive
3	Male	64	HPSCC	4	2	IV	Poor	Positive
4	Male	68	HPSCC	4	1	IV	Well-moderately	Negative
5	Male	65	HPSCC	3	1	III	Poor	Negative
6	Male	69	HPSCC	3	2	IV	Poor	Positive

LSCC, laryngeal squamous cell carcinoma; HPSCC, hypopharyngeal squamous cell carcinoma.

The unexpected opposite trend in tissue and plasma EVs suggests that circulating plasma EVs miR-99a-5p may be a potential biomarker for the detection of HNSCC.

### miR-99a-5p as a Biomarker for Early HNSCC Detection

**FIGURE 3:**
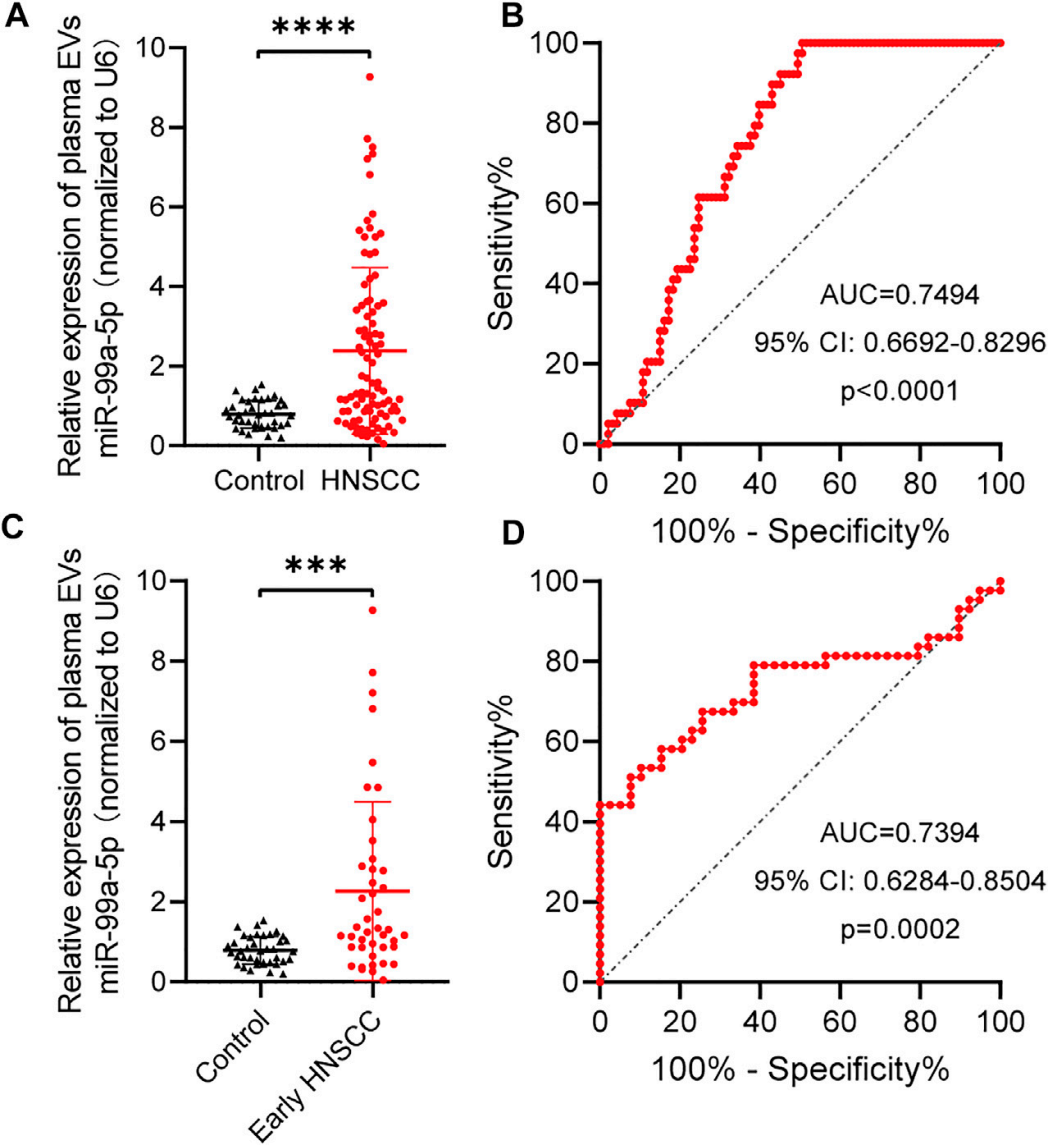
miR-99a-5p as a biomarker for early HNSCC detection. **(A)** miR-99a-5p expression in HNSCC patients (*n* = 93) compared with that in benign disease patients (*n* = 39) detected by qRT-PCR. U6 serves as an internal control. Mann–Whitney test, ****p < 0.0001. **(B)** Diagnostic potential of plasma EVs miR-99a-5p in HNSCC patients. The area under the ROC curve was 0.7494 (95% CI: 0.6692 to 0.8296; *p* < 0.0001). **(C)** miR-99a-5p expression in early HNSCC patients (*n* = 43) compared with that in benign disease patients (*n* = 39) detected by qRT-PCR. U6 serves as an internal control. Mann–Whitney test, ***p = 0.0001. **(D)** Diagnostic potential of plasma EVs miR-99a-5p in early HNSCC patients. The area under the ROC curve was 0.7394 (95% CI: 0.6284 to 0.8504; *p* = 0.0002). The results are expressed as mean ± SD.

To further confirm the hypothesis previously proposed, we explored the diagnostic value of plasma EVs miR-99a-5p as a minimally invasive HNSCC diagnostic biomarker. We isolated plasma EVs from HNSCC patients (n = 93) and benign disease patients (*n* = 39) and assessed the miR-99a-5p expression level by qRT-PCR. We found that miR-99a-5p expression levels were significantly higher in plasma EVs from HNSCC patients (median: 1.445) than in those from benign disease patients (median: 0.7687) (*p* < 0.0001) (Figure 3A). Detailed clinical and pathological data on 93 HNSCC patients are presented in Table 3. Overall, patients with high plasma EVs miR-99a-5p expression were significantly younger (*p* = 0.0129) and had a lower p16 positive status in pathological samples (*p* = 0.0134) than those with low expression. No further significant associations were found between miR-99a-5p levels in HNSCC plasma EVs and clinicopathological features (T stage, lymph node metastasis, clinical stage, tumor diameter, or pathological differentiation).

**TABLE 3:** Correlation between plasma EVs miR-99a-5p and clinicopathologic characteristics in 93 HNSCC patients.

Parameter	All patients		High expression		Low expression		*p* value
	*n* = 93	%	*n* = 38	%	*n* = 55	%	
Age							***0.0129*** *
≤60	22	23.7	14	36.8	8	14.5	
>60	71	76.3	24	63.2	47	85.5	
Gender							0.0650
Female	3	3.2	3	7.9	0	0	
Male	90	96.8	35	92.1	55	100	
Smoking history							0.9185
No	25	26.9	10	26.3	15	27.3	
Yes	68	73.1	28	73.7	40	72.7	
Drinking history							0.4071
No	34	36.6	12	31.6	22	40	
Yes	59	63.4	26	68.4	33	60	
Hypertension							0.5451
No	50	53.8	19	50	31	56.4	
Yes	43	46.2	19	50	24	43.6	
Diabetes							0.1906
No	83	89.2	36	94.7	47	85.5	
Yes	10	10.8	2	5.3	8	14.5	
p16							***0.0134*** ^†^
Negative	70	75.3	34	89.5	36	65.5	
Positive	23	24.7	4	10.5	19	34.5	
T stage							0.2769
T1+T2	43	46.2	15	39.5	28	50.9	
T3+T4	50	53.8	23	60.5	27	49.1	
Lymph node metastasis							0.6989
Negative	29	31.2	11	28.9	18	32.7	
Positive	64	68.8	27	71.1	37	67.3	
Clinical stage							0.7305
I + II	18	19.4	8	21.1	10	18.2	
III + IV	75	80.6	30	78.9	45	81.8	
Maximum tumor diameter (MTD)							0.8957
<3 cm	35	37.6	14	36.8	21	38.2	
≥3 cm	58	62.4	24	63.2	34	61.8	
Differentiation							0.3476
High + High-moderate	81	87.1	35	92.1	46	83.6	
Moderate + moderate-low	12	12.9	3	7.9	9	16.4	

*p value was tested from Chi-square test.

†*p* value was tested from Fisher’s exact test.

To assess the potential value of plasma EVs miR-99a-5p for the diagnosis of HNSCC, ROC curves were constructed to distinguish HNSCC patients from benign disease patients (Figure 3B). The AUC was 0.7494 (95% CI: 0.6692–0.8296; *p* < 0.0001). When the optimal cut-off value was 0.8537, the sensitivity and specificity were 61.54% (0.4590–0.7511) and 75.27% (0.6562–0.8292), respectively.

Given the current lack of effective biomarkers for early HNSCC, we further explored whether plasma EVs miR-99a-5p could be a biomarker for early HNSCC detection. We found that early HNSCC patients (*n* = 43) had higher expression levels of miR-99a-5p in plasma EVs than benign disease patients (*p* = 0.0001) (Figure 3C). Plasma EVs miR-99a-5p expression level was able to discriminate early HNSCC from benign disease patients with an AUC of 0.7394 (95% CI: 0.6284–0.8504; *p* = 0.0002). Furthermore, using the cut-off value mentioned above, the sensitivity and specificity were 79.07% (0.6479–0.8858) and 61.54% (0.4590–0.7511), respectively (Figure 3D).

Collectively, plasma EVs miR-99a-5p levels distinguished early HNSCC and were associated with HPV infection status of patients with HNSCC.

## Discussion

Plasma EVs present several advantages in carrying cargo from tumor tissue that make them reliable and specific for use as diagnostic tools [5]. These include being stable molecules that can be easily detected in peripheral circulation plasma and the fact that their expression has been correlated with clinicopathological features, thus offering promise as prognostic and predictive biomarkers [21]. We previously demonstrated that plasma EVs TGFβ1 is higher in HNSCC patients than in control patients, and is associated with clinicopathological features of HNSCC patients. More importantly, plasma EVs TGFβ1 expression could clearly distinguish between HNSCC and control patients with higher diagnostic efficiency than total TGFβ1 expression in plasma [16].

Several studies have reported that plasma EVs miRNAs are potential biomarkers for the diagnosis and survival prediction of HNSCC patients [22]. A recent study found that increased plasma EVs miR-491-5p levels were associated with poor overall survival and disease-free survival in HNSCC patients. Plasma EVs miR-491-5p was able to distinguish HNSCC patients with sensitivity and specificity of 46.6% and 100%, respectively [23].

In the current study, we first confirmed the high expression of miR-99a-5p in para-tumor tissue *via* miRNA sequencing and further verified it using TCGA data. The upregulation of miR-99a-5p in para-tumor tissue is also consistent with previous studies [19, 24]. Notably, the expression of miR-1246 was consistent in tumor tissue and plasma EVs in our previous study [17]. However, we found that miR-99a-5p expression in tumor tissue and plasma EVs showed an opposite trend in HNSCC patients. This unexpected opposite trend of miR-99a-5p in tissue and plasma was also reported by Garrido-Cano [21] and Torres [25] in breast cancer and endometrioid endometrial carcinoma. Moreover, other investigators have demonstrated that several other miRNAs have opposite expression patterns in tissues and peripheral blood [26, 27, 28]. These suggests that circulating miR-99a-5p, especially in plasma EVs, may be a potential biomarker for the detection of HNSCC.

MiR-99a-5p has been reported as a tumor suppressor in cancer. Tamai et al. showed that miR-99a-5p induced cellular senescence in gemcitabine-resistant bladder cancer cells by targeting SMARCD1 [11]. Sun et al. found that miRNA-99a-5p suppresses cell proliferation, migration, and invasion by targeting isoprenylcysteine carboxylmethyltransferase (ICMT) in oral squamous cell carcinoma [13]. Based on these findings and our data, we speculated that HNSCC cells might release the tumor suppressor miR-99a-5p into the peripheral circulation through EVs to maintain their survival. Selective EVs packaging and the release of miRNAs have been reported to occur in cancer cells as a means of eliminating tumor suppressors [29, 30, 31]. However, the processes by which miRNAs are selectively packed in plasma EVs remain poorly understood [32, 33]. Our results reinforce the concept of selective release, which may explain the discrepancy between tissue and plasma EVs.

Further, in this study, we observed a relationship between the miR-99a-5p expression level and HPV infection status. Overall, HPV positive was associated with more favorable clinical outcomes in HNSCC patients [2]. Although the miR-99a-5p expression level was not associated with clinical outcomes, patients with high miR-99a-5p expression in tissue had a significantly more p16 positive status in pathological samples than patients with low miR-99a-5p expression. By contrast, patients with high miR-99a-5p expression in plasma EVs had less p16 positive status in pathological samples than that of patients with low expression, which is consistent with the opposite trend we observed in tissue and plasma EVs. Further research is needed to clarify the relationship between miR-99a-5p and HPV infection status.

There were several limitations to this investigation. First, validating the diagnostic efficiency of plasma EVs miR-99a-5p with multi-centric validation research is required. Our study lacked prognostic data, as all cases were newly diagnosed (from October 2019 to October 2021). Therefore, the prognostic value of plasma EVs miR-99a-5p could not be evaluated in this study. Second, although we ruled out the possibility that plasma EVs are derived from platelets and immune cells, whether they are completely derived from HNSCC remains to be discussed. This is also one of the bottlenecks restricting plasma EVs as an indicator of tumor diagnosis and prognosis, which needs more efforts to solve. Third, the reasons for the opposite trend of miRNA in HNSCC tumor tissues and plasma EVs have not been studied in detail. More studies should focus on the key pathways and provide an explanation for the opposite trend.

To the best of our knowledge, this is the first study to evaluate the clinical application of plasma EVs miR-99a-5p as a diagnostic biomarker in HNSCC patients. Our study demonstrates two important clinical findings. First, we found that miR-99a-5p is downregulated in HNSCC tissue and is associated with HPV infection status. Second, we identified the dynamic change of miR-99a-5p between tissue and plasma EVs, and confirmed that plasma EVs miR-99a-5p is a potential diagnostic biomarker for early HNSCC patients.

Collectively, we primarily evaluated the value and provided evidence of plasma EVs miR-99a-5p as a biomarker for early HNSCC. This may help improve early HNSCC detection.

## Data Availability

The datasets generated during and/or analysed during the current study are available from the corresponding authors on reasonable request.
